# Effect of Force of Microneedle Insertion on the Permeability of Insulin in Skin

**DOI:** 10.1177/1932296813519720

**Published:** 2014-05

**Authors:** Karmen Cheung, Tao Han, Diganta Bhusan Das

**Affiliations:** 1 Department of Chemical Engineering, Loughborough University, Loughborough, Leicestershire, UK

**Keywords:** insertion force, microneedles, microtome, permeability, rheometer, viscoelasticity

## Abstract

Many experiments conducted in the literature have investigated the effect of microneedles (MNs) on insulin permeation across skin. There are also a number of articles that deal with the effect of MN insertion force in skin. However, there is little known on quantifying the relationship between the effect of MN insertion force and the amount of insulin permeated for given MNs. This issue is addressed in this article. MNs of 1100 µm and 1400 µm are used to conduct in vitro permeability experiments on porcine skin, using insulin. Histological images of MN treated skin are obtained from a microtome and the viscoelastic properties of the skin sample are measured using a rheometer. An in-house insertion force device is utilized that can reproducibly apply a specified force on MNs for a set period of time using compressed air. It is deduced that when porcine skin was pretreated with an applied force of 60.5 N and 69.1 N, the resultant amount of insulin permeated was approximately 3 µg and 25 µg over a 4-hour period for the MNs used. The amount of MN force applied to porcine skin was shown to be related to the amount of insulin permeated. An increase in insertion force increase the amount of insulin permeated. It was also demonstrated that using insufficient force may have reduced or prevented the amount of insulin passing through the skin, regardless of the geometry of the MNs.

Microneedle (MN) treatment is a well-developed technology in transdermal drug delivery.^1,2^ It can be employed either on its own or in corporation with other techniques.^3^ The MNs are aimed at minimizing mass transfer distance between the drugs and the blood microcirculation by physically creating micron-scale channels in skin which let the drug molecules to transport through the skin over a shorter distance. The performance of the MN patch on enhancing drug delivery is based on 3 main factors. First, the inherent design of the MN patch which include the geometry (length, radius, shape) and the number of needles (MNs density) are important as they directly affect the drug permeability and the insertion behaviour.^2,4,5^ Second, for a certain type of MN patch, the force applied on the patch will determine its insertion behavior and, hence, its performance. The insertion force is used to overcome a number of force components, as discussed in detail by Olatunji et al.^4^ An appropriate force can help the MNs to overcome the resistance of the skin,^4^ where the resistance is typically determined by the viscoelastic property of the skin. It is known that the top layer, that is, the stratum corneum (SC), is the dominating skin layer that governs the overall viscoelasticity of the skin^5,6^ and provides the maximum resistance to MN insertion into the skin. Third, the molecular weight of the drug molecule is important as it indicates if it can passively pass through the SC. Large molecules may need sufficient penetration depth to have a reasonable permeation rate. Passive diffusion is applicable only for molecules that are traditionally smaller than 500 Da,^6,7^ that is, the typical molecular cutoff of the SC.

Insulin has a molecular weight of 5.8 kDa and is widely used in the treatment of diabetes. Insulin cannot pass through skin passively due to its large molecular size but research shows that it can be delivered through skin with the treatment of different types of MNs. These MNs include dissolving, solid and hollow MNs and are applied both in vivo and in vitro.^8-10^ An in vivo study on rats has successfully shown a great amount of permeation of insulin.^8,9^ But, in an in vitro study, while the porcine skin has been chosen as typical samples for experiment, the permeated amount of insulin is much less than that in rat skin sample due to the higher thickness and stronger mechanical properties of the porcine skin.^9,11^ Because porcine skin is more structurally similar to human skin, the permeability enhancement for insulin in porcine skin poses great relevance in research. No doubt, the design and pattern of the MNs patch can directly affect the drug permeation results.^10,12^ But another crucial factor in the enhancement of permeation is the force applied on the patch as mentioned earlier. A sufficient force is needed to ensure that the MNs are properly penetrated into the skin instead of only buckling the skin sample. Full penetration of MNs will need even higher force to achieve which may give more permeation of drug molecules. It is expected that normal thumb pressure will be applied while inserting MNs into skin, but different humans are expected to exert different amounts of force while inserting MNs. Numerous researches have been conducted to study MN insertion force and its relationship to various parameters, such as tip radius,^11,13^ pain upon penetration,^12,14^ and penetration depths.^13,15^ However, there have been little or no studies conducted on the direct relationship between MN insertion forces and the permeation of large proteins, such as insulin. We address this issue and attempt to eliminate this knowledge gap by exploring this relationship by relating the total insertion force acting on 2 different MN patches and the in vitro permeation of insulin in porcine skin. As the insertion behavior depends on the viscoelasticity of the skin, we determine the properties of these skin samples used in this work. Drug permeation is then related to the amount of force applied for a given MN patch. It seems that to reach the expected insulin delivery rate, a relatively high MN insertion force is required for the skin samples used. We discuss these in more detail in the latter part of the article.

To achieve the purpose of this study, 2 different MN systems with varying lengths are used, which were done so as to confirm that the trend of results obtained from 1 particular MN length is observed for another length of MN. It is not the objective of this study to compare directly the performance of 1 MN array with the performance of another as the geometry of the MNs is different. The chosen MNs maintain continuity of some of our previous studies.^3,16-18^

## Materials and Methods

### Materials

Actrapid insulin (manufactured by Novo Nordisk, Kalundborg, Denmark) was purchased from a local pharmacy shop. A reverse phase high performance liquid chromatography (RP-HPLC) instrument (Agilent Series 1100, Cheadle Cheshire, UK) was used to determine insulin concentration. Two reagents, acetonitrile and trifluoroacetic acid (TFA), were used as the mobile phases for the HPLC analysis. They were obtained from Fisher Scientific UK Ltd (Loughborough, UK). A Kinetex (length: 100 mm, internal diameter: 4.6 mm) column equipped with a Security Guard column (Phenomenex, Inc, Macclesfield, UK) was used to quantify the samples containing insulin. A manual Franz diffusion cell (FDC; Logan Instruments Corporation, Franklin Township, NJ) was used to conduct the permeation studies with and without MNs. Deionized water purified using a Millipore Elix System (Billerica, MA) was used for all FDC experiments. The skin samples used for the permeation studies were taken from porcine ears that were purchased from a local abattoir. The detailed preparation of the skin samples was the same as mentioned by Han and Das.^18^ Two commercial MN patches, namely, AdminPatch Array 1200 MN and AdminPatch Array 1500 MN array (AdminMed, Sunnyvale, CA) were used to pretreat the porcine skin. These patches have been proved to help passage of large molecules through the skin.^18^ The MN patches contain MNs of different dimensions, and therefore they are well suited for use in this work as they allow us to relate the MN dimensions, force of insertion, and insulin permeability. There are 43 individual needles on the 1100 µm MNs patch and 31 individual needles on the 1400 µm MNs patch. The main characteristics of the MNs are shown in Table 1.

**Table 1.** The Characterizations of the MN Array.

Parameter	AdminPatch MN 1500	AdminPatch MN 1200
Number of MNs	31	43
Length (µm)	1400	1100
Width (µm)	580	460
Thickness (µm)	100	100
Space between MNs (A) (µm)^a^	1970	1410
Space between MNs (B) (µm)^a^	3000	2610
Space between MNs (C) (µm)^a^	1550	1650

a Refer to Figure 5E for spacing orientation.

An experimental rig (device) was built in house to apply a variation of MN forces onto porcine skin sample. All the components of this device were purchased from RS Components Ltd (Corby, UK). The device is discussed in more detail in the next subsection.

Methylene blue was used for staining experiment and was purchased from Sigma-Aldrich (Gillingham, Dorset, UK). In combination with optical microscopes, a cryotome instrument (Bright Instruments Co Ltd, Huntingdon, UK) was used to ascertain depth profiles (histology) of the pretreated and non-pretreated skin samples. The viscoelasticity of the skin samples were measured by using a rheometer from TA Instrument (New Castle, DE).

### MN Insertion Force Device

**Figure 1.**
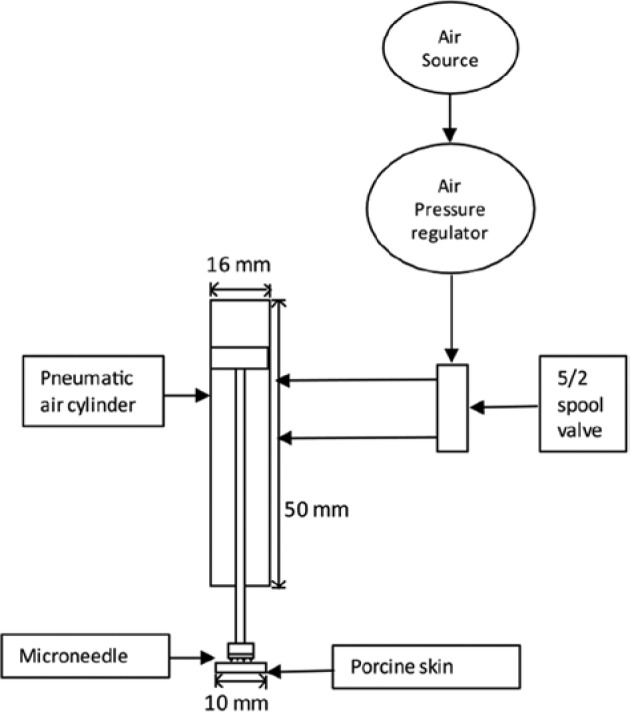
Schematic diagram of the force device equipment setup, which is used to apply a specified force on MNs for a given time duration.

This article has used an in-house system that has been manufactured specifically to provide a reproducible force on MNs for insertion onto skin samples (Figure 1). The following are used to construct the device: a double acting roundline cylinder, a 16 × 50 mm pneumatic air cylinder, an air pressure regulator, and a directional control valve (5/2 spool valve). The developed system operates as follows. Compressed air pressure is used to activate the pneumatic cylinder in the system, which acts like a piston. The imposed air pressure can be recorded that mimics the load applied on to the MN. The amount of compressed air running through the system is controlled by an air pressure regulator, and it is operated by a spool valve lever. The system is fitted with a directional control valve that has 3 settings. When the lever is in the central position (neutral position), the internal shaft of the pneumatic cylinder is free moving as the spool valve is open and under atmospheric pressure. In this position the MNs can be attached to the MN holder and then placed on top of the porcine skin with no excessive force. When the lever is moved to the “on” position, the regulated compressed air is channeled to the top of the pneumatic cylinder. This forces the air piston to move down as the air flows through and maintains a constant force onto the porcine skin for a required time. When the lever is moved to the “off” position, the regulated compressed air is channeled to the bottom of the pneumatic cylinder, which forces the air piston to move up to the top, as the air flows through. The imposed force and the MNs are perpendicular to the skin sample at the point where the MN contacts the sample.

This enables us to define that the applied force is used to penetrate the MNs in the skin samples. Subsequently, the force (ie, air pressure over active area) exerted by the insertion device on the MN patch is calculated using the following equation:

$$F=\frac{p\pi \left(d_{1}^{2}-d_{2}^{2}\right)}{4},$$

where F is the force exerted (N), p is the air pressure (N/m^2^), $d_{1}$ is the bore piston diameter (m), and $d_{2}$ is the piston rod diameter (m).^19^

### Permeability Measurements Using Franz Diffusion Cell

**Figure 2.**
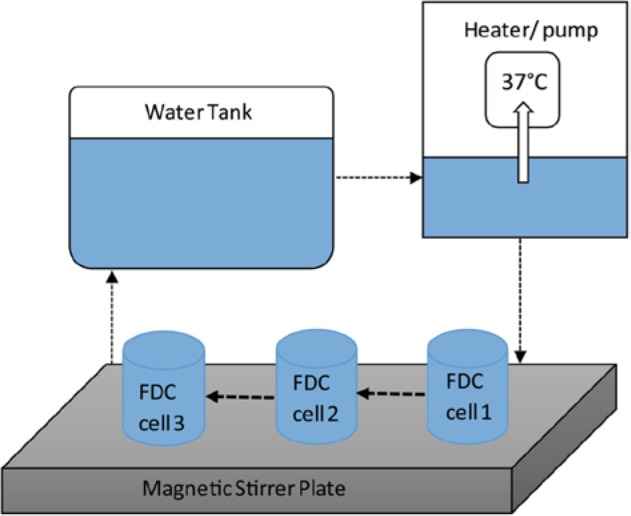
Franz diffusion cells system consisting of 4 main components, namely, a heater/pump, water tank, magnetic stirrer plate, and diffusion cells.

An FDC apparatus (Figure 2) was used to measure the insulin permeability in skin.^18^ A total of 70 µg of insulin containing solution was placed into the donor chamber and samples extracted from the receiving compartment at time points of 1, 2, 3, and 4 hours. The procedures for conducting the permeation experiments are the same as detailed by Han and Das,^18^ and therefore they are not discussed in this article.

### MN Insertion Into Porcine Skin

The porcine skin in this study was not stretched when a force was applied. This was due to the various porcine thicknesses used, which would add variability in calculating the percentage increase of the original size. This would be inadequately controlled and therefore may lead to variation in the tension of each sample. Therefore, it would be hard to keep the same tension under in vivo conditions.

The force device (Figure 1) was used to apply a range of pressures onto the surface of the porcine skin (0.10, 0.20, 0.30, 0.35, and 0.40 MPa) which are converted to the total forces of 17.3 N, 34.6 N, 51.8 N, 60.5 N, and 69.1 N, respectively. The forces were calculated based on a pneumatic cylinder used in the experimental setup. The equation used for the calculation is shown in Equation 1. The forces were chosen based on experimental results, in which a force increase was applied to allow a permeation of drug. As the 1500 and 1200 AdminPatch MN patches consist of 31 and 43 MNs on each patch, the force acting on 1 individual MN is approximately 2.2 N and 1.6 N, respectively, assuming that the applied force is aligned in the same direction as the MNs and perpendicular to the skin. Pre-excised porcine skin cut into 1.5 cm^2^ pieces stored in a petri dish was placed below the MN. The MNs attached to the piston were then aligned on top of the porcine skin. The spool valve was set to the on position, where a constant force of MN was inserted into porcine skin for 5 minutes. The skin sample was then removed and placed onto the receptor chamber of a diffusion cell.

### Method of Analyzing Insulin Concentration Using HPLC

The concentration of the sample from the FDC experiment was analyzed by RP-HPLC. The diode array detector (DAD) was set at 214 nm.^20^ A gradient method was adopted with eluent A of 0.1% TFA in water and B of 0.1% TFA in acetonitrile, with a mobile phase ratio of A:B, 95:05 to 05:95. The sample size of each injection was 10 µL. The temperature was set to 30°C. The flow rate was set to 1 ml/min. A complete RP-HPLC run took approximately 10 minutes with a down time of 2 minutes between each run.

### Sectioning of Skin Samples Using Cryotome

**Figure 3.**
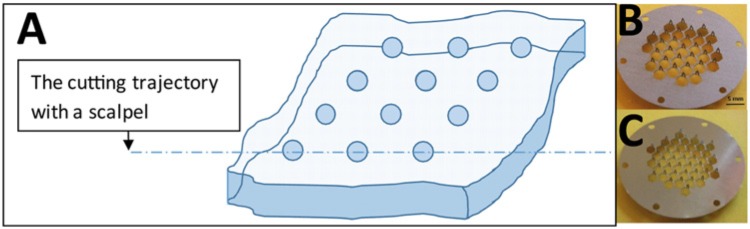
(A) The MN pretreated skin sample is cut through in a row using a scalpel. (B) Image of AdminPatch Array 1500. (C) Image of AdminPatch Array 1200.

A depth profile (histology) of the pretreated (MN) and non-pretreated porcine skin was prepared using cryotome. The pretreated skins were subjected to 1100 µm and 1400 µm MNs with the insertion forces applied for 5 minutes. In this article 69.1 N insertion force was chosen to obtain a clearer cross-sectional image of the skin sample and the holes created by the MN. Methylene blue dye was diluted with water to prepare a 50/50 (v/v) solution. The diluted dye solution was carefully poured onto the top surface of the skin sample and washed off after 3 min. Subsequently, the stained sample was cut into a 2 × 2 cm^2^ pieces. The skin sample was then placed into the cryotome machine for 24 hours. The frozen sample was perpendicularly cut through 1 row of MN holes using a surgical scalpel, which is shown in Figure 3A. Figures 3B and 3C depict the AdminPatch Array 1500 MN and AdminPatch Array 1200. During this procedure, the cutting side was attached to the mold’s wall to make sure that the section showed a real depth profile of MN holes that could be observed.

**Figure 4.**
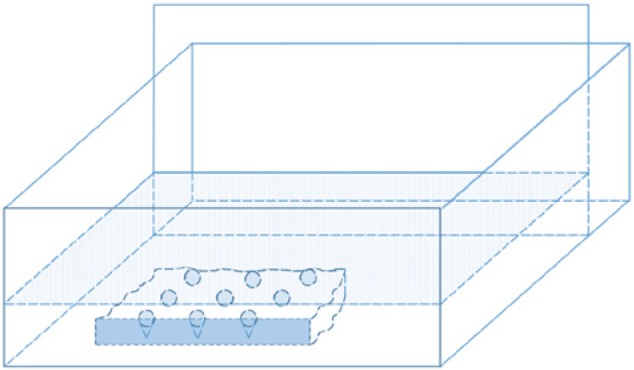
The setup of the mold for microtome sectioning.

The mold consisted of a rectangular plastic base and a glass baffle plate. The glass baffle plate was removed after the specimen was frozen. After the removal of the baffle plate, the frozen specimen could easily be removed from the base mold while keeping its structural integrity. The mold was filled with embedding medium (Bright Cryo-m-Bed, Huntingdon, UK) to a 1 mm depth and kept in the freezer until the gel was completely solidified. The skin sample was then mounted onto the frozen layer with its cutting side sticking to the front inner wall of the mold. More embedding medium was filled until the whole skin sample was submerged. The whole mold was placed in the liquid nitrogen tank for flash freezing. The setup of the molding section is shown in Figure 4.

The temperature in the microtome was set to −20°C. The angle of the blade was set to a normal cutting position of 15 degrees. The antiroll guide plate was set parallel to the cutting bevel and has a 50 µm gap in between. The top surface of the guide plate was just above the blade’s edge. The thickness of the slices was set to 15 µm. The specimen was glued onto the object hold using embedding medium and left in the chamber of the microtome until it was tightly bound to the holder.

The whole system and the sample required 1 hour to reach the optimum sectioning temperature, which was −20°C. The sample was cut with a reasonable speed to ensure its integrity. A room temperature glass slide was then applied on to the specimen. After the specimen melted onto the slide, it was carefully removed from 1 side to avoid any movement of the sample.

### Viscoelasticity of Skin Samples

To characterize the viscoelasticity of the skin samples, the storage and loss modulus of the skin as well as the viscosity were determined. The storage and loss moduli are recorded at a volume strain of 1% with an increasing angular frequency from 6 to 474 rad/s.^21^ The storage (G’) and loss moduli (G") shows the elastic and viscous properties of the material, respectively. They can be defined as,

$$G^{\prime }=\frac{\tau_{0}}{\gamma_{0}}\cos\delta$$

$$G^{\prime \prime }=\frac{\tau_{0}}{\gamma_{0}}\cos\delta$$

where τ_0_ is the oscillatory stress, $\gamma_{0}$ is the strain (1%), and δ is the phase shift (10-20 degrees). The equations show that at a certain strain both the storage and loss moduli have proportional relationships with the stress. It indicates that if the storage modulus is high, there will be more resistant force from the material against the external forces. The rheometer is set to an oscillating mode, and the geometry of the plate is plain with teeth to make sure that there is no movement between the plate and skin sample during the test. The viscosity of the skin sample is also calculated from the loss modulus, and it can be related to the shear rate using the following equations,

$$\tau =\eta \dot{\gamma }$$

$$\eta =\frac{G"}{\omega }$$

where τ is the shear stress, η is the viscosity, ω is the angular frequency, and $\dot{\gamma }$ is the shear rate.

## Results and Discussion

### Characterization of Porcine Skin

**Figure 5.**
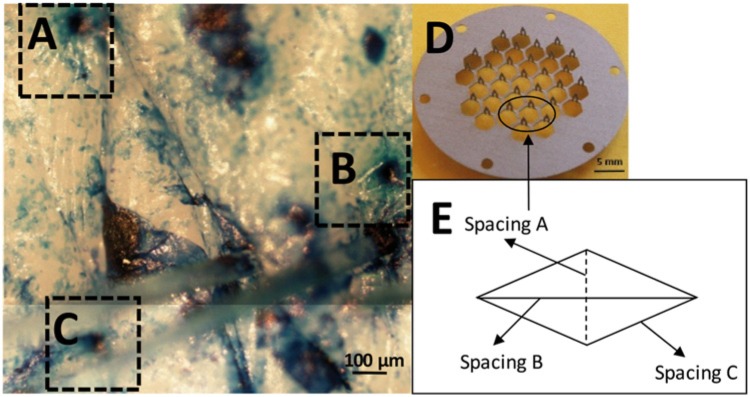
(A, B, C) Three insertion holes created from 3 individual MNs using AdminPatch 1500 MN array using 69.1 N insertion force. The diameters of the holes are 57 µm, 50 µm, and 64 µm, respectively, calculated using ImageJ. (D) Photographic image of AdminPatch Array 1500. (E) Schematic diagram of the AdminPatch Array 1500. It shows spacing between 4 equidistant MNs, spacing A: 1.97 mm, spacing B: 3.00 mm, and spacing C: 1.55 mm, calculated using ImageJ.

Staining experiments were conducted on porcine skin to visualize that the needle insertion create holes in the skin samples. Figures 5A, 5B, and 5C depict an image of the porcine skin when it has been punctured by the 1500 AdminPatch MN (Figure 5D). Figure 5E shows a schematic diagram of the spacing between 4 equidistant MNs. The results show that the needles have successfully pierced into the skin. The dimensions of the holes are shown to be A-57 µm, B-50 µm, and C-64 µm.

**Figure 6.**
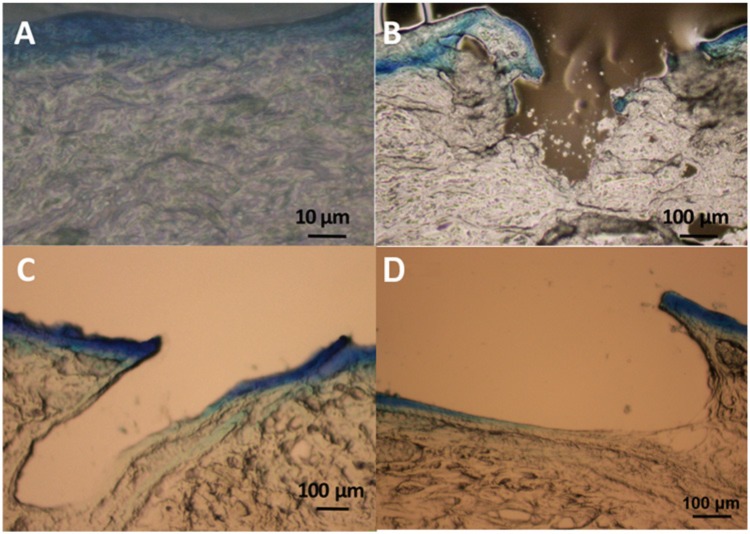
(A) Microscopic images taken of cryotome porcine skin (without MN). (B) Porcine skin pretreated with 1400 µm MN with a force of 69.1 N. (C) Porcine skin pretreated with 1100 µm MN with a force of 69.1 N. (D) porcine skin pretreated with 1100 µm MN with a force of 60.5 N.

As mentioned earlier, cryotome was used to visualize the cross section of porcine skin with and without MN pretreatment. Methylene blue dye was applied to the surface to see what effect MNs have on the SC. Figure 6A shows the cross section of porcine skin with no pretreatment of MNs which, as expected, shows that the SC is not disrupted. Figure 6B shows a view where the SC has been disrupted when a force of 69.1 N was applied using the 1400 µm MNs. Figure 6C shows the same force being applied using the 1100 µm MN patch, the depth is affected as it is shown that the holes created by the MNs are much more shallow. The 1100 µm MNs are smaller in sizes which make them easier to be bent. This MN patch also has a much higher MN density as compared to the 1400 µm patch. So it increases the insertion difficulty as well as it makes it less able to disrupt the skin sample. Figure 6D shows the results using the 1100 µm microneedles under 60.5 N. It seems to indicate that the skin sample is more like being stretched and scraped rather than being penetrated by the MNs patch. Due to the geometry of the MNs, the needles are pushed horizontally by the resistance of the skin instead of allowing them to pierce vertically into the sample under 60.5 N force.

The shape of the hole is also more integrated because of the size of the needle and shows that during insertion the MN may have had bent.

### Viscoelasticity of Skin Samples

**Figure 7.**
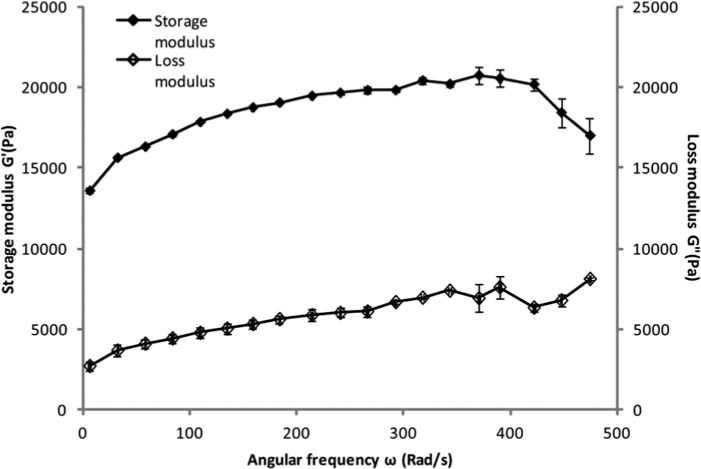
The average storage (G’) and loss (G") moduli of the skin samples with an increasing angular frequency (n = 2).

The importance of considering viscoelastic properties during MN insertion has been discussed earlier, for example, by Olatunji et al.^4,5^ The variation in the viscoelasticity of the skin is likely to affect the insertion behavior of the MNs, and hence it is best to consider the specific skin samples and their properties. As such, we discuss the viscoelasticity of the skin samples used in this work. The results in Figure 7 show that the skin samples are predominantly elastic as G’>>G”.

The figure shows that the storage modulus is over 15000 Pa while the loss modulus is only around 5000 Pa. It also means that a high resistance to external forces is likely to be offered by the skin sample. This has been somewhat confirmed by our difficulty in inserting the chosen MN patch, particularly with the high needle density version (AdminPatch Array 1200).

**Figure 8.**
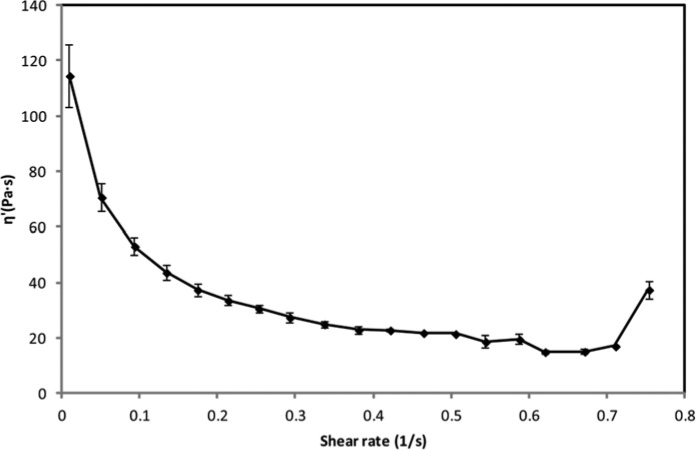
The average viscosity (η) of the skin samples with an increasing shear rate (n = 2).

The viscosity is calculated using the loss modulus at every angular frequency. As the strain is set to 1%, the shear rate will be rising when the angular frequency is increasing. The resultant relationship between the viscosity and the shear rate is shown in Figure 8.

### Permeability of Insulin

A variation of forces applied on MNs was conducted on porcine skin in vitro to ascertain the amount of insulin permeated when the MNs were used for pretreatment. Porcine skin that was not treated with MNs was used as a control. Insulin Actrapid is a 5808 kDa molecule, which is considerably larger than 500 Da required for passive diffusion through the SC. Therefore no insulin is expected to permeate the skin when conducting passive diffusion experiments.

**Figure 9.**
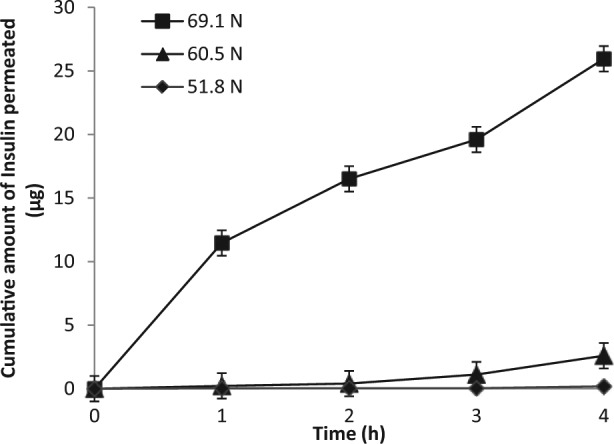
Cumulative amount of insulin (µg) permeated with a MN insertion force 51.8 N, 60.5 N, and 69.1 N for over 4 hours (n = 3). The amounts of insulin permeated for MN insertion forces of 17.3 N and 34.6 N and passive diffusion were undetectable.

The amount of insulin permeated for passive diffusion and MN insertion forces of 17.3 N and 34.6 N was almost undetectable. Therefore no results have been reported in this article. This may be due to the skin only being buckled or slightly pierced, therefore sufficient pathways cannot be created to let the insulin molecules pass through. Figure 9 illustrates the amount of insulin permeated when MN penetration forces of 51.8 N, 60.5 N, and 69.1 N were used to pretreat the porcine skin samples. The total amount of the insulin used for each diffusion experiment was 70 µg and the ratio of the insulin in the receiving compartment and donor compartment was shown to increase from 0.2% to 37.1%. The results show that after 4 hours the amount of insulin permeated was approximately 3 μg and 25 μg, respectively, for 60.5 N and 69.1 N insertion forces, but were negligible when an insertion force of 51.6 N was applied. There was almost an 8-fold increase in the amount permeated using the larger insertion force. More important, it confirms that an increase in the insertion force results in an increase in the amount of insulin permeated. The lack of insulin permeated when insertion forces less than 51.8 N were used could be the result of the “bed of nails” effect.

Although the SC layer is likely to have been disrupted, the high density MNs may not have been able to pierce through the whole epidermis due to the elastic properties of the skin, greatly affecting the permeation of insulin. This resulted in a lower concentration of insulin, compared to the less dense MN.

**Figure 10.**
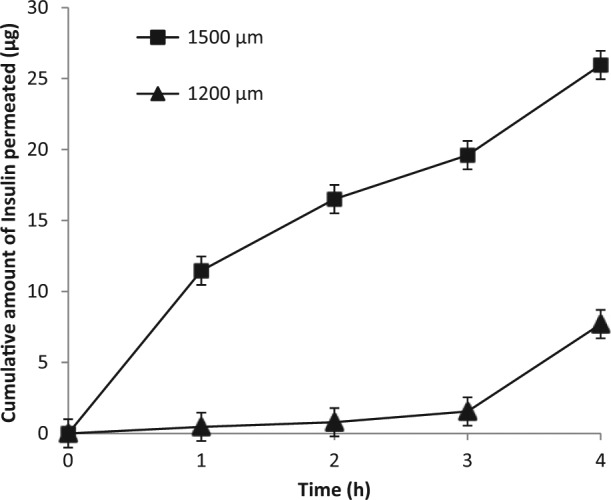
Cumulative amount of insulin (µg) permeated with a MN insertion force of 69.1 N for over 4 hours for 1500 and 1200 AdminPatch MNs (n = 3). The amounts of insulin permeated for MN insertion forces of 17.3 N and 34.6 N and, passive diffusion were undetectable.

Figure 10 illustrates the amount of insulin permeated when 1400 µm and 1100 µm MNs of penetration force 69.1 N was used to pretreat porcine skin. After 4 hours, the amount of the permeated insulin with the 1400 µm patch pretreatment was approximately 3 times higher than the 1100 µm patch. The ratio of total received insulin increases from 11% to 37.1%. It indicates that the geometry of the MN is also a crucial factor that can affect the insulin permeation. The length of the MNs is directly related to the diffusion pathway and is the main factor that determines the permeation of insulin. Our results also demonstrate that the insertion behavior of the MNs, and therefore the hole size are affected by the viscoelastic property of the skin and the needle density on the patch. Although the insertion force is high, a higher density of the MNs can result in a shorter insertion depth than expected which leads to a lower permeation.

## Conclusion

MNs have been used to increase the permeability of countless drug molecules. The amount of MN force applied to porcine skin was related to the amount of insulin permeated. From the permeability experiments it is shown that an increase in insertion force increased the amount of insulin permeated through porcine skin. An insufficient force may not help the insulin to pass through the skin regardless of the geometry of the MNs. But, on a detectable level, the length of the MN and the force applied on the MNs are important factors that can greatly affect the permeation.

As a scope for future work, it must be stated that more study on the effects of insertion force of MNs on the drug permeability should be conducted. For example, as an industrial application, the MNs used in this work could be used as a potential for sustained drug release, and if so further studies on the clinical applications of these types of MNs should be conducted.
